# Transcriptomic insights into the molecular mechanism for response of wild emmer wheat to stripe rust fungus

**DOI:** 10.3389/fpls.2023.1320976

**Published:** 2024-01-03

**Authors:** Jing Ren, Liang Chen, Jian Liu, Bailing Zhou, Yujie Sha, Guodong Hu, Junhua Peng

**Affiliations:** ^1^ Shandong Key Laboratory of Biophysics, Institute of Biophysics, Dezhou University, Dezhou, China; ^2^ Key Laboratory of Plant Germplasm Enhancement and Specialty Agriculture, Wuhan Botanical Garden, Chinese Academy of Sciences, Wuhan, China; ^3^ Spring Valley Agriscience Co., Ltd, Jinan, China

**Keywords:** transcriptome, stripe rust, molecular mechanism, wild emmer wheat, transcription factor

## Abstract

**Introduction:**

Continuous identification and application of novel resistance genes against stripe rust are of great importance for wheat breeding. Wild emmer wheat, *Triticum dicoccoides*, has adapted to a broad range of environments and is a valuable genetic resource that harbors important beneficial traits, including resistance to stripe rust caused by *Puccinia striiformis* f. sp. *tritici* (*Pst*). However, there has been a lack of systematic exploration of genes against Pst races in wild emmer wheat.

**Methods:**

Genome-wide transcriptome profiles were conducted on two wild emmer wheat genotypes with different levels of resistance to (*Pst* (DR3 exhibiting moderate (*Pst* resistance, and D7 displaying high (*Pst* resistance). qRT-PCR was performed to verify findings by RNA-seq.

**Results:**

A higher number of DEGs were identified in the moderately (*Pst*-resistant genotype, while the highly (*Pst*-resistant genotype exhibited a greater enrichment of pathways. Nonetheless, there were consistent patterns in the enrichment of pathways between the two genotypes at the same time of inoculation. At 24 hpi, a majority of pathways such as the biosynthesis of secondary metabolites, phenylpropanoid biosynthesis, phenylalanine metabolism, and alpha-Linolenic acid metabolism exhibited significant enrichment in both genotypes. At 72 hpi, the biosynthesis of secondary metabolites and circadian rhythm-plant pathways were notably and consistently enriched in both genotypes. The majority of (*WRKY, MADs *, and *AP2-ERF * families were found to be involved in the initial stage of response to *Pst* invasion (24 hpi), while the *MYB, NAC, TCP*, and *b-ZIP* families played a role in defense during the later stage of *Pst* infection (72 hpi).

**Discussion:**

In this present study, we identified numerous crucial genes, transcription factors, and pathways associated with the response and regulation of wild emmer wheat to *Pst* infection. Our findings offer valuable information for understanding the function of crucial *Pst*-responsive genes, and will deepen the understanding of the complex resistance mechanisms against *Pst* in wheat.

## 1 Introduction

Wheat is one of the most important crops in the world, providing more than 20% of total human food calories (Zhao et al., 2020). Stripe rust (also called yellow rust), caused by *Puccinia striiformis* f. sp. *tritici* (*Pst*), is generally considered one of the most devastating diseases of wheat all over the world (Schwessinger, 2017; Wu et al., 2023). In an infected field, this disease can cause significant reductions in both the yield and quality of wheat. In extreme situations, it can result in yield losses of up to 100% (Peng et al., 1999). Although stripe rust can be controlled through the timely application of fungicides, growing resistant cultivars has proven to be the most effective, economical and environmentally safe means of controlling the stripe-rust disease. Discovering key genes for stripe rust resistance can provide important elements for breeding resistant cultivars.

To date, a total of 86 correctly named stripe rust resistant (*Yr*) genes (*Yr1-Yr86*) and over 100 *Yr* genes with temporary names have been identified in wheat and its wild relatives (Zhu et al., 2023). Among these, certain wheat resistance genes, such as *Yr9* (Liu et al., 2008), *Yr15* (Yaniv et al., 2015), and *Yr26* (Wang et al., 2008), have been extensively utilized in wheat breeding programs. However, the continuous emergence of new virulence races within *Pst* populations has rendered most of the stripe rust resistance genes ineffective (Poland et al., 2009; Zhang et al., 2021; Zhao et al., 2022). Chinese wheat varieties, such as Nanda 2419, Ganmai 8, Zhongliang 11, Nongda 139, and Chuanmai 42 become susceptible due to the development of new virulent races during these years (Kang et al., 2015; Zhao et al., 2022). Therefore, there is a pressing need to identify and utilize novel genes that exhibit exceptional resistance against the emerging virulent *Pst* races.

The greatest hope for future crop genetic improvement lies in exploiting the gene pools of the wild relatives of crop plants (Peng et al., 1999). Hence, wild emmer wheat, *T. dicoccoides*, the progenitor of all cultivated wheats, is particularly promising for stripe rust resistance. Wild emmer wheat is believed to have originated in the northeastern Israel and the Golan Heights and adaptively diversified throughout the Fertile Crescent, across a variety of ecological conditions (Nevo, 2002). It has adapted to a broad range of environments and is a valuable genetic resource that harbors important beneficial traits, including a potential source of genes for stripe rust resistance (Nevo, 2002; Fu et al., 2009; Peng et al., 2011). Broad-spectrum stripe rust resistance genes *Yr36* (Fu et al., 2009) and *Yr15* (Klymiuk et al., 2018), derived from wild emmer wheat, have been cloned. And also, several resistance genes, including *Yr30/Sr2* (Singh et al., 2005; Liu et al., 2017), *Yr35/Lr52* (Dadkhodaie et al., 2011) and *YrH52* (Peng et al., 2000) derived from wild emmer wheat, have been mapped onto various chromosomes. Thus, wild emmer wheat represents one of the best hopes for genetic improvement of wheat stripe rust resistance.

RNA sequencing (RNA-seq) is a highly sensitive and comprehensive strategy for transcriptome analysis (Wang et al., 2021). It has been widely used in recent years for identifying genes and understanding the mechanisms underlying plant-pathogen interactions (Xu et al., 2011; Wang et al., 2021; Cai et al., 2023; Wang et al., 2023). Wang et al. (2023) recently employed mutagenesis and transcriptome sequencing to successfully clone the leaf rust resistance gene *Lr9*. Based on RNA-seq analysis, the underlying mechanisms of transcription factor *BZR2* confer resistance to wheat stripe rust were revealed by Bai et al. (2021). Although previous studies have documented several wheat resistance genes against stripe rust, there has been a lack of systematic exploration of genes against *Pst* races in wild emmer wheat using RNA-Seq. The objectives of this study were to comprehensively analyze the transcriptome profiles of leaves from two wild emmer genotypes with different *Pst* resistance and identify crucial genes and pathways associated with the response and regulation of wild emmer wheat to *Pst* infection, as well as to elucidate the underlying molecular mechanisms involved in the response to strip rust. The findings will offer significant insights for enhancing the resistance of wheat cultivars against prevalent *Pst* races through molecular breeding techniques.

## 2 Materials and methods

### 2.1 Plant materials and *Pst* inoculation

In both field and greenhouse conditions, a total of 350 wild emmer wheat accessions were inoculated with a mixture of the prevalent Chinese *Pst* races comprising CY31, CY32, and CY34. Among these, two wild emmer wheat genotypes, namely DR7 and DR3, with different level of *Pst* resistance were used for identifying genes in response to *Pst* infection. Infection types (ITs) were assessed 20 days after inoculation using a previously established 0 to 9 scale (Chen and Line, 1992). Following *Pst* infection, DR7 exhibited a notable level of resistance against *Pst* races, as indicated by an IT value of 0 (**Supplementary Figure S1**). Whereas, DR3 demonstrated a moderate level of resistance against *Pst* races. More specifically, the DR3 genotype displayed chlorotic and necrotic areas in the leaves, accompanied by light sporulation. The IT value for DR3 was measured at 3-4, with a disease severity ranging from 20-30% (**Supplementary Figure S1**). Wheat plants were cultivated and subjected to *Pst* inoculation for RNA-Seq analysis, following the methodology outlined by Kang and Li (1984). Leaf samples were collected at three different time periods, i.e., 0, 24, and 72 hours post-inoculation (hpi). Sampling from each period was conducted with three biological replicates. The samples collected at 0 hpi were labeled as CK. The samples collected from DR7 at 24 and 72 hours after inoculation were labeled as DR7_24 and DR7_72, respectively, while the corresponding samples from DR3 were labeled as DR3_24 and DR3_72. All fresh samples were promptly frozen in liquid nitrogen and stored at -80 ° until RNA extraction.

### 2.2 RNA extraction, library construction and sequencing

The total RNA was extracted from the leaf samples collected at the specific time points using the Trizol reagent (Invitrogen) following the manufacturer’s protocol. Subsequently, the extract was treated with DNaseI to eliminate genomic DNA. The quantity and quality of the total RNA were assessed using an Agilent 2100 Bioanalyzer system. Following this, a total of 18 cDNA libraries were constructed and sequenced using the Illumina HiSeqTM 2000 system (Illumina, San Diego, CA, USA) at the Novogene Bioinformatics Technology Co., Ltd. (Beijing, China).

### 2.3 Transcripts assembly and sequencing alignment

The 18 libraries produced raw reads, which underwent a series of preprocessing steps to eliminate low-quality reads, adaptor sequences, and reads containing poly-N. Quality indicators, including Q20 (%), Q30 (%), error rate (%), and GC (%) content, were then calculated for further analysis. The clean reads were aligned to the wild emmer wheat reference genome using the HISAT2 software (https://daehwankimlab.github.io/hisat2/, version 2.2.1) (Kim et al., 2019). The wild emmer wheat genome WEW_v2.0, available at https://www.ncbi.nlm.nih.gov/genome/13332?genome_assembly_id=444209, was used as the reference. The assembly of transcripts was conducted utilizing the StringTie software (https://ccb.jhu.edu/software/stringtie/#top, version 1.3.4) (Pertea et al., 2016), followed by merging of spliced transcripts from each sample using the Cuffmerge software (version 2.2.1) (Trapnell et al., 2010). Subsequently, the transcripts were categorized into known and novel transcripts through comparison with the database. The quantification of gene expression was determined using the fragments per kilobase of transcript sequence per million base pairs sequenced (FPKM) methodology (Trapnell et al., 2010).

### 2.4 Differential expression analyses of genes

For the purpose of conducting differential expression analysis, the samples were subjected to pairwise comparisons between different time points within the same genotype. Specifically, the comparisons included DR3_24h *vs* DR3_CK, DR3_72h *vs* DR3_CK, DR7_24h *vs* DR7_CK, and DR7_72h *vs* DR7_CK. The DESeq2 method (Love et al., 2014) was employed for this analysis. The selection of significant differential expression genes (DEGs) was based on stringent criteria of *Padj <*0.05, |log_2_Ratio| ≥ | 1, and FPKM ≥ 1. To cluster the differentially expressed genes, hierarchical clustering was performed using the FPKM value of each sample.

### 2.5 GO terms and KEGG enrichment analyses of DEGs

The GOseq (Young et al., 2010) was used to conduct Gene Ontology (GO) enrichment analysis on differentially expressed genes. These genes were categorized into three GO categories: biological process (BP), cellular component (CC) and molecular function (MF). Additionally, the DEGs underwent enrichment analysis using the Kyoto Encyclopedia of Genes and Genomes (KEGG) (Kanehisa et al., 2017) to identify *Pst*-responsive metabolic pathways and associated candidate genes. The hypergeometric test was used to determine significantly enriched GO terms and KEGG pathways, with a corrected *P*-value threshold of <0.05.

### 2.6 Identification and analysis of transcription factors

Transcription factors (TFs) were predicted using the iTAK (version 18.12) (http://itak.feilab.net/cgi-bin/itak/index.cgi) and assigned to different families (Zheng et al., 2016). The coexpression networks were constructed using the WGCNA package version 1.42 (Langfelder and Horvath, 2008). TFs with FPKM values > 1 were used for the WGCNA co-expressed network analysis. The modules were obtained by employing the automatic network construction function blockwiseModules with default settings. The network hub was defined as a highly connected gene within a network that exhibited high intra-modular connectivity.

### 2.7 Validation of mRNAs by qPCR analysis

To ensure the reliability and validity of the RNA-seq findings, a total of 20 DEGs were randomly selected for quantitative real-time PCR (qRT-PCR) analysis. The same RNA samples that were used to for RNA-seq were also utilized for qRT-PCR. The gene-specific primers (listed in **Supplementary Table S1**) required for qRT-PCR analysis were designed using Primer Premier 5.0 software (Premier Biosoft International, Palo Alto, CA, USA). Total RNA was extracted from the leaf samples following the procedure outlined in Section 2.2. Subsequently, first-strand cDNA was synthesized using M-MLV reverse transcriptase (Promega) and an oligo (dT) primer, following the manufacturer’s instructions. This cDNA was then utilized as a template for RT-PCR analysis. All qRT-PCR reactions were performed in triplicate, and the 2^−ΔΔCT^ method was employed to calculate relative expression levels (Pfaffl, 2001). The Tublin gene served as an internal reference for normalizing the expression data in the qRT-PCR analysis. The correlation between RNA-Seq and qRT-PCR was assessed using SPSS 22.0 software (SPSS Institute Ltd., Armonk, NY, USA).

## 3 Results

### 3.1 Results and quality assessment of transcriptome sequencing

A total of 2,881,302,618 raw reads were generated from the 18 libraries, as indicated in **Supplementary Table S2**. After the removal of low-quality reads, adapter sequences, and reads with poly-N, a total of 2,873,356,960 clean reads were obtained (**Supplementary Table S2**). The “Q20” and “Q30” values exceeded 97% and 93% respectively, while the GC content ranged from 43.61% to 46.99% (**Supplementary Table S2**). These statistics suggest that the sequencing data exhibited high quality. Subsequently, the clean reads were aligned to the wild emmer wheat reference genome using the HISAT2 software (Kim et al., 2019). On average, 95.23% of the reads were successfully aligned with the reference genome, and 75.22% of these reads were unique (**Supplementary Table S2**).

The quantification of gene expression levels was achieved using normalized FPKM (Trapnell et al., 2010). The FPKM data underwent rigorous testing to assess correlations between biological replicates, with all obtained Pearson correlation coefficients exceeding 0.91 (**Supplementary Figure S2A**). Notably, the results of principal component analysis (PCA) demonstrate a strong clustering pattern among the biological replicates for each sample, as depicted in **Supplementary Figure S2B**. Taken together, these findings prove the high reproducibility and reliability of our experimental data.

**Figure 1:**
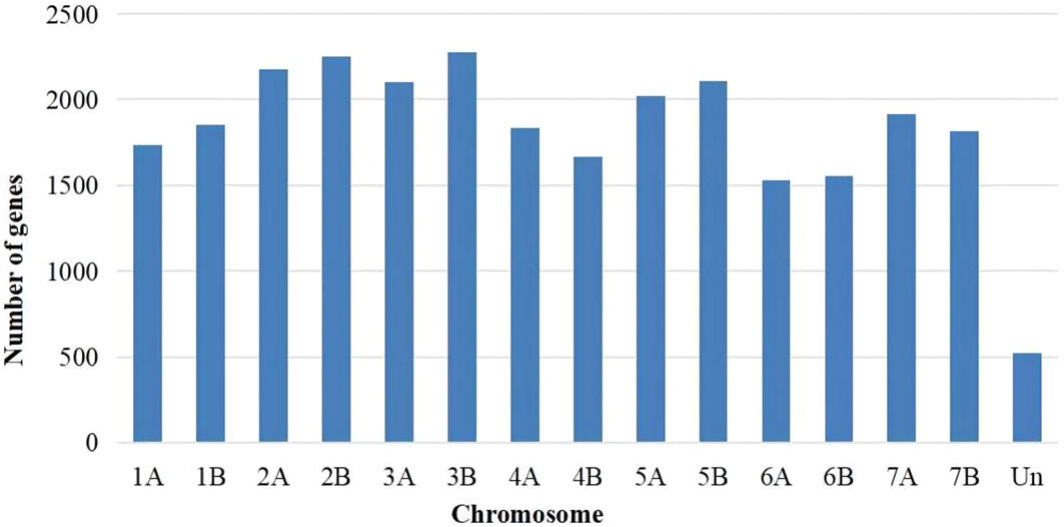
Genomic distribution of genes with FPKM ≥ 1.

Genes that exhibited expression were determined based on FPKM values > 1. Utilizing Cufinks software (Trapnell et al., 2010), a total of 27,355 annotated expressed genes were identified across the eighteen samples. The distribution of these 27,355 expressed genes across the chromosomes is depicted in **Figure 1**.The distribution of genes across the 14 chromosomes was relatively uniform, with the lowest number observed on chromosome 6A (1,528 genes) and the highest number observed on chromosome 3B (2,275 genes).

### 3.2 Characterization of differentially expressed genes in response to *Pst* infection

**Figure 2:**
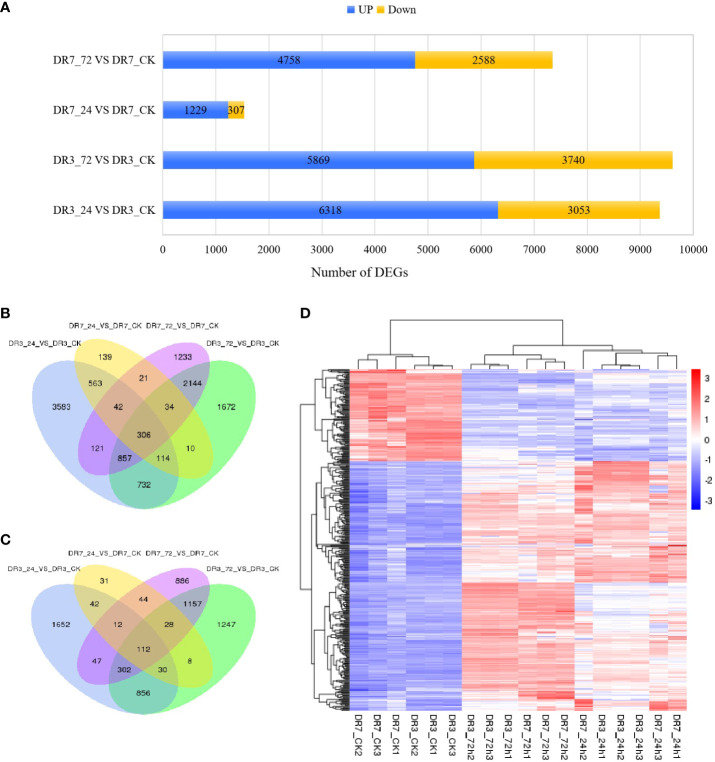
Identification and characterization of differentially expressed genes (DEGs) between pairs of libraries based on criteria of *Padj* < 0.05, |log_2_Ratio| ≥1 and FPKM >1. **(A)** The numbers of DEGs detected across the four comparison groups (DR3_24h *vs* DR3_CK, DR3_72h *vs* DR3_CK, DR7_24h *vs* DR7_CK, and DR7_72h *vs* DR7_CK); **(B)** Venn diagram of up-regulated genes across the four comparison groups; **(C)** Venn diagram of down-regulated genes across the four comparison groups; **(D)** Hierarchical cluster analysis of 418 common *Pst*-responsive genes across all samples. Red color indicates that the gene is highly expressed in the sample, and the blue color indicates that the gene expression is low.

Based on the criteria of |log_2_Ratio| ≥ |1|, *Padj* < 0.05, and FPKM ≥ 1, a total of 16,659 DEGs were obtained when comparing the 24 hpi and CK samples, as well as the 72 hpi and CK samples, in the two accessions (**Figures 2B, C**). In the DR3 genotype, a total of 9,371 genes exhibited differential expression at 24 hpi, with 6,318 genes showing upregulation and 3,053 genes showing downregulation. Similarly, at 72 hpi, 9,609 genes displayed differential expression, with 5,869 genes upregulated and 3,740 genes downregulated (**Figure 2A**). In the DR7 genotype, 1,536 genes exhibited differential expression at 24 hpi, with 1,299 genes upregulated and 307 genes downregulated. At 72 hpi, 7,346 genes displayed differential expression, with 4,758 genes upregulated and 2,588 genes downregulated (**Figure 2A**). The DR3 genotype exhibited a greater proportion of differentially expressed genes compared to the DR7 genotype at both 24 and 72 hpi, suggesting that the inoculation of *Pst* may exert a more substantial regulatory impact on the genotype with moderate resistance to *Pst* than those with high resistance to *Pst* (**Figure 2A**).

Venn diagram analyses were conducted to identify the presence of overlapping and unique genes in various comparison groups. In the DR3 genotype, a total of 8,407 DEGs were specific to this particular genotype. Of these, 4,067 DEGs were unique to the 24 hpi, while 2,607 DEGs were unique to the 72 hpi. Moreover, 1,773 DEGs were found to be shared between the 24 hpi and 72 hpi. In the DR7 genotype, a total of 1,772 DEGs were exclusively expressed. Among these, 148 DEGs were unique to the 24 hpi, while 1,562 DEGs were specific to the 72 hpi. Additionally, 62 genes were found to be common to the two time points (**Figures 2B, C**). Significantly, a total of 418 common DEGs (306 upregulated and 112 downregulated) were identified in response to *Pst* infection across four comparisons (DR3_24h *vs* DR3_CK and DR3_72h *vs* DR3_CK, and DR7_24h *vs* DR7_CK and DR7_72h *vs* DR7_CK) (**Figures 2B, C**). The identification of these 418 common DEGs is of great importance in understanding the defense mechanisms against stripe rust. Their presence suggests the existence of conserved regulatory pathways that are activated by *Pst* infection in both genotypes. Notably, further investigation of these DEGs revealed consistent expression patterns between DR7 and DR3 at the same *Pst*-inoculation time point (**Figure 2D**). Although there was minor variation observed among the three replications, such as DR7 at 24 hpi, the differentially expressed genes at the same *Pst*-inoculation time point were well clustered and effectively distinguished from other *Pst*-inoculation time point.

### 3.3 GO and KEGG enrichment analyses of differentially expressed genes

**Figure 3:**
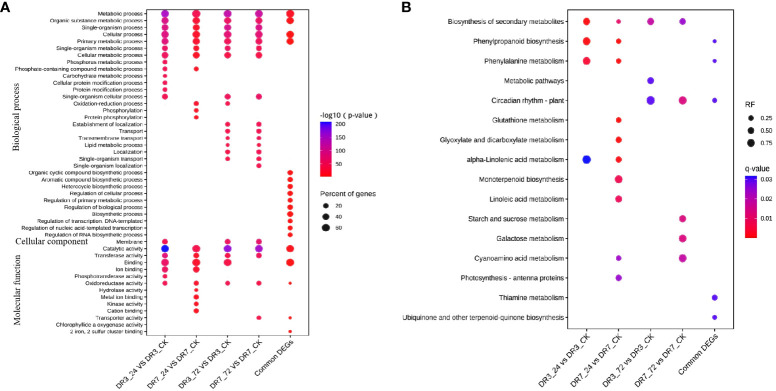
GO and KEGG pathway enrichment analysis of differentially expressed genes in response to stripe rust. **(A)** GO enrichment analysis of DEGs; only the significantly terms were showed. **(B)** KEGG enrichment analysis of DEGs; only the significantly enriched pathways were showed.

The DEGs from each comparison groups (DR3_24h *vs* DR3_CK, DR3_72h *vs* DR3_CK, DR7_24h *vs* DR7_CK, and DR7_72h *vs* DR7_CK) and 418 common DEGs across all four comparison groups were subjected to GO analysis to predict their biological function in response to *Pst*-inoculation (**Figure 3A**). The top 20 enriched terms for each comparison groups are displayed in **Figure 3A**. A great number of DEGs were found to be involved in BP function and MF function in this study. Specifically, metabolic process, organic substance metabolic process, single-organism process, cellular process, primary metabolic process, single-organism metabolic process, and cellular metabolic process in the BP, as well as catalytic activity, transferase activity, and oxidoreductase activity in the MF, were commonly enriched across all comparison groups (DR3_24h *vs* DR3_CK, DR3_72h *vs* DR3_CK, DR7_24h *vs* DR7_CK, and DR7_72h *vs* DR7_CK). Importantly, 418 common DEGs were also dominantly enriched in metabolic process, organic substance metabolic process, cellular process, and primary metabolic process in the BP and catalytic activity, binding, and oxidoreductase activity in the MF (**Figure 3A**). In addition, it was observed that at 24 hpi, the DR7 and DR3 genotypes exhibited common enrichment in three terms: phosphate-containing compound metabolic process, binding, and ion binding. Similarly, at 72 hpi, both genotypes showed common enrichment in several terms, including single-organism cellular process, establishment of localization, transport, transmembrane transport, lipid metabolic process, localization, single-organism transport in the MF category, and membrane in the CC category (**Figure 3A**). These findings suggest that genes associated with these categories may have significant roles in the defense against *Pst* infection in both DR7 and DR3 genotypes.

To identify the metabolic pathways activated in response to *Pst*-inoculation, we searched the DEGs against KEGG database. The significant enriched pathways for each comparison groups are presented in **Figure 3B**. In the DR3 genotype, several pathways including biosynthesis of secondary metabolites, phenylpropanoid biosynthesis, phenylalanine metabolism, and alpha-Linolenic acid metabolism were found to be significantly enriched at 24 hpi (**Figure 3B**). The pathways of biosynthesis of secondary metabolites, secondary metabolites and circadian rhythm - plant were significantly enriched at 72 hpi (**Figure 3B**). A greater abundance of pathways exhibited enrichment in DR7 compared to DR3. At 24 hpi, the significantly enriched pathways in the DR7 include biosynthesis of secondary metabolites, secondary metabolites, circadian rhythm-plant, glutathione metabolism, glyoxylate and dicarboxylate metabolism, alpha-Linolenic acid metabolism, monoterpenoid biosynthesis, cyanoamino acid metabolism, and photosynthesis antenna proteins (**Figure 3B**). Similarly, at 72 hpi, the significantly enriched pathways in the DR7 included biosynthesis of secondary metabolites, circadian rhythm - plant, starch and sucrose metabolism, galactose metabolism, and cyanoamino acid metabolism (**Figure 3B**). These results suggest that the DR7 genotype, which exhibits a high level of resistance to *Pst*, has a more complex regulatory networks when exposed to *Pst* infection, as compared to the genotype with moderate resistance (**Figure 3B**). Notably, the analysis revealed that 418 DEGs shared among the four comparison groups were also significantly enriched in the pathways of phenylpropanoid biosynthesis, phenylalanine metabolism, circadian rhythm-plant, thiamine metabolism and ubiquinone (**Figure 3B**).

### 3.4 Transcription factors in response to *Pst* infection

Transcription factors (TFs) play a significant regulatory role in the wheat response to *Pst* infection (Bai et al., 2021). A total of 298 differentially expressed TFs from 9 families were identified, according to the criteria of |log_2_Ratio| ≥ |1|, Padj < 0.05, and FPKM ≥ 1. These nine TF families identified were *NAC*, *WRKY*, *MYB*, *bHLH*, *AP2/ERF*, *MADS*, *bZIP*, *HY*, and *TCP*. Of these families, the *WRKY* family was the most abundant in the response of wild emmer wheat to *Pst* infection. As shown in **Table 1**, the majority of these TFs were upregulated, and DR3 had a higher proportion of differentially expressed TFs in comparison to DR7 at both the 24 and 72 hpi.

**Table 1:** Identification and characterization of differentially expressed transcription factors in four comparison groups based on the criteria of *Padj* < 0.05, |log_2_Ratio| ≥1 and FPKM > 1.

Transcription factors	DR3_24 *vs* DR3_CK		DR3_72 *vs* DR3_CK		DR7_24 *vs* DR7_CK		DR7_72 VS DR7_CK	
	Up	Dn	Up	Dn	Up	Dn	Up	Dn
NAC	24	8	25	9	13	1	12	9
WRKY	41	3	16	6	13	0	7	9
MYB	27	5	33	12	6	0	27	10
bHLH	11	6	16	6	3	1	10	6
AP2/ERF	32	0	15	4	14	0	8	10
MADS	8	1	6	4	1	0	2	9
bZIP	9	1	11	2	2	1	11	2
HY	0	0	6	0	0	0	7	0
TCP	0	1	4	4	0	0	3	2
Total	152	25	132	47	52	3	87	57

**Figure 4:**
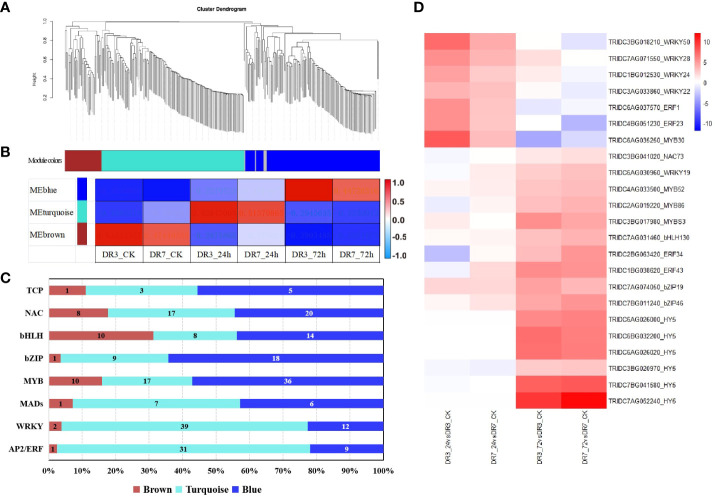
WGCNA of differentially expressed transcription factors (TFs). **(A)** Cluster dendrogram of TFs based on expression levels after *Pst* infection. Each branch represents a gene and each color below represents a gene co-expression module. **(B)** Correlation of transcription factor expression patterns at different *Pst*-inoculation time points (0, 24 and 72 h). The color bar indicates correlation from low (blue) to high (red). **(C)** Distribution of transcription factor families in different WGCNA modules. Brown: 0 hpi; Turquoise: 24 hpi; Blue: 72 hpi. Each color in the visual representation signifies a co-expression module, while the numerical values correspond to the quantity of transcription factors present within each module. **(D)** A heatmap showing fold-changes of hub genes identified across the four comparisons. The color gradient represents log_2_fold-change between the *Pst*-infection group and the control group with red color referring to the upregulated genes and blue color referring to the downregulated genes.

To analyze the co-expression and correlation networks of these differentially expressed TFs, we conducted weighted correlation network analysis (WGCNA) as depicted in **Figure 4**. The findings indicate that the differentially expressed TFs can be categorized into three distinct co-expression modules, indicated with different colors (brown, turquoise and blue) (**Figure 4A**). Further analysis indicates that these three modules (colored brown, turquoise and blue) exhibit a strong correlation with *Pst*-inoculation time (0 hpi, 24 hpi, and 72hpi) (**Figure 4B**). Significantly, TFs in both DR3 and DR7 genotypes exhibit similar expression patterns during the same time of *Pst*-inoculation, but with higher expression levels in DR3 compared to DR7. The majority of TFs belong to the turquoise module (131) and blue module (120), indicating that the highest expression of most TFs occurred at 24 hpi and 72hpi, respectively. Notably, there was family-specific expression observed at different time points (**Figure 4C**). The majority of *WRKY*, *AP2-ERF*, and *MADs* reached their highest expression level at 24 hpi, whereas all *HY5* and over half of *MYB, TCP*, and *bZIP* reached their peak expression at 72 hpi.

Within each module, hub genes were identified, including *MYB3, MYB52, MYB86, ERF34, ERF43, bZIP19, bZIP46, bZIP(HY5), WRKY19, NAC73*, and bHLH130 in the blue module. Similarly, the turquoise module was found to have *WRKY22, WRKY24, WRKY28, WRKY50, MYB30, NAC2, NAC17, ER1*, and *ERF23* as its hub genes.

### 3.5 Validation of RNA-seq analysis by quantitative real-time PCR

To evaluate the reliability of DEGs obtained through RNA-seq analysis, a random selection of 20 DEGs were subjected to qRT-PCR analysis (**Supplementary Table S1**), with Tublin as the reference gene for normalization. The findings demonstrated a good agreement with the gene expression pattern derived from RNA-Seq, although there were minor discrepancies in the log2 Fold change values. A highly significant correlation (R^2^ = 0.9094, n = 80; correlation coefficient of qRT-PCR log_2_FC versus RNA-seq log_2_FC) between the RNA-seq and qRT-PCR data was observed (**Supplementary Figure S3**). This outcome unequivocally validates the reliability of the DEGs obtained from RNA-seq analysis in this study.

## 4 Discussion

### 4.1 Potential value of wild emmer wheat in wheat breeding for stripe rust resistance

Evaluation of resistance against stripe rust can help us understand the application potential of wild emmer wheat germplasm in wheat breeding programs for resistance against the stripe rust disease. In the present study, we systematically assessed the stripe rust response of a germplasm collection including 350 wild emmer wheat accessions. The germplasm, collected from Near East Fertile Crescent, represent the natural distribution of wild emmer wheat. A total of 49 accessions identified were completely immune to the prevalent stripe rust races, and 6 moderately resistant under both the field and greenhouse conditions. Hence, the resistant accessions screened are the useful core germplasm for mining resistant genes for wheat genetic improvement. Among these, two wild emmer wheat genotypes, namely DR7 and DR3, with different level of *Pst* resistance were used for identifying genes in response to *Pst* infection.

### 4.2 Global patterns of transcription in response to *Pst*-infection in wild emmer wheat

The emergence of new and highly virulent races of *Pst* poses a significant threat to global wheat production, making it imperative to identify novel *Pst*-resistant genes in order to enhance wheat resistance against this devastating stripe rust disease (Kang et al., 2015; Schwessinger, 2017). RNA-seq analysis is the most promising method for elucidating the molecular mechanisms underlying plant-pathogen interactions, including the roles of genes, pathways, and transcription factors involved (Cui et al., 2017; Bai et al., 2021; Xu et al., 2023). In the present study, we conducted RNA-seq analysis on two genotypes with different levels of resistance to *Pst*. A total of 16,659 DEGs were identified in the two genotypes. Notably, a greater number of DEGs were observed in DR3 (9,371 at 24 hpi; 9,609 at 72 hpi) compared to DR7 (1,536 at 24 hpi; 7,346 at 72 hpi) (**Figure 2A**). A higher number of DEGs were detected in DR3 at 24 hpi, suggesting that global gene expressions were more quickly initiated in the moderately *Pst-*resistant genotype than those in the highly *Pst-*resistant genotype, when they were exposed to *Pst-*infection. However, the analysis of KEGG showed that more pathways were enriched in DR7 than in DR3 (**Figure 3B**). This suggests that more metabolic pathway and more complex molecular mechanisms were triggered in the highly *Pst-*resistant genotype to against stripe rust. Notably, significantly enriched pathways had similar patterns at the same inoculation time between the two genotypes. At 24 hpi, most pathways including biosynthesis of secondary metabolites, phenylpropanoid biosynthesis, phenylalanine metabolism, and alpha-Linolenic acid metabolism were commonly enriched in both genotypes (**Figure 3B**). While biosynthesis of secondary metabolites and circadian rhythm-plant were identified as the commonly enriched pathways at 72 hpi (**Figure 3B**). Importantly, these 418 commonly identified DEGs across the four comparison groups were significantly enriched in the pathways of phenylpropanoid biosynthesis, phenylalanine metabolism, circadian rhythm-plant, thiamine metabolism, and ubiquinone. These findings shed light on the potential significance of these pathways in conferring wheat resistance against *Pst* infection, as depicted in **Figure 3B**.

### 4.3 Phenylpropanoid biosynthesis and phenylalanine metabolism could play a role in wheat resistance against *Pst* during the early stage of infection

The phenylpropanoid pathway, which is responsible for the biosynthesis of many secondary compounds, plays a crucial role in plant defense against pathogens (Govender et al., 2017; Dong and Lin, 2021). Previous study has shown that the activation of phenylpropanoid biosynthesis and phenylalanine metabolism pathways occurred in wheat after 24 hpi (Hao et al., 2016). Similarly, our findings indicate that these pathways were significantly enriched at 24 hpi in both the genotypes, suggesting their potential importance in conferring wheat resistance against *Pst* during the initial phase of infection.

**Figure 5:**
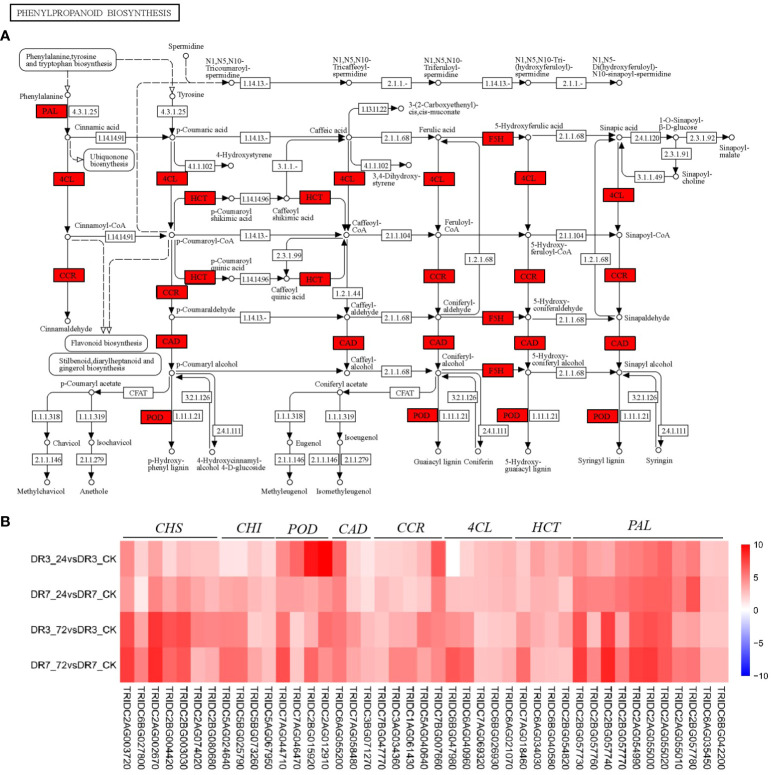
Phenylpropanoid biosynthesis pathway and its expression pattern in response to *Pst* infection in the two wild emmer wheat genotypes, DR7 and DR3. **(A)** The phenylpropanoid biosynthesis pathway map. The regulatory network is constructed based on the expression levels of known genes involved in this pathway. The red color indicated an increase in expression levels at 24 hpi in both DR7 and DR3 genotypes. **(B)** A heatmap showing fold-changes of *Pst*-responsive genes in phenylpropanoid biosynthesis across the four comparisons. The color gradient represents log2 of fold-change between the *Pst*-infection group and the control group with red color referring to the upregulated genes and blue color referring to the downregulated genes. PAL, phenylalanine ammonia-lyase; HCT, shikimate Ohydroxycinnamoyl transferase; 4CL, 4-coumarate-CoA ligase; CCR, cinnamoyl-CoA reductase; CAD, cinnamyl-alcohol dehydrogenase; POD,peroxidase; CHI, chalcone isomerase; CHS, chalcone synthase.

Previous studies have demonstrated that phenylalanine ammonia-lyase (PAL) functions as the gateway enzyme in phenylpropanoid metabolism and plays a key role in the response to various environmental stimuli, including pathogen infection (Yuan et al., 2019; Pant et al., 2021; Liu et al., 2023b). Here we show that the expression of the majority of *PAL* genes in wild emmer wheat is significantly increased in response to *Pst* infection in the two accessions. Although the enrichment factors of phenylpropanoid biosynthesis and phenylalanine metabolism were significantly higher in the moderately *Pst*-resistant genotype of DR3 compared to the highly *Pst-*resistant genotype of DR7, the up-regulated expression fold of the *PAL* gene in DR3 was significantly lower than that in DR7 (**Figure 5B**). This finding is consistent with a previous study by Chen et al. (2013), which showed the involvement of *PAL* in phenylpropanoid biosynthesis in both the *Yr39*-mediated high-temperature adult-plant (HTAP) and *Yr5*-mediated all-stage resistances of wheat. Recent report showed that *TaPAL* contributes to wheat resistance to *Pst* by regulating lignin and phenol synthesis, which was verified by the virus-induced gene silencing (VIGS) technique (Liu et al., 2023b).

### 4.4 The continuous accumulation of secondary metabolites contributed to the resistance to *Pst* in wild emmer wheat

In plants infected with *Pst*, the induction of multiple secondary metabolite pathways was observed. PAL catalyzes the polymerization of lignin, flavonoids, hydroxycinnamic acid, phenolics, and anthocyanin, among other secondary metabolites (**Figure 5A**). These metabolites play crucial roles in plant defense mechanisms (Dong and Lin, 2021; Pant et al., 2021; Liu et al., 2023b). Of particular importance is lignin, which serves as a component of secondary cell walls and contributes to defense against various pathogens (Xu et al., 2011; Yuan et al., 2019). The deposition of lignin strengthens the cell wall and provides a physical barrier against pathogen spread (Xu et al., 2011; Yuan et al., 2019). Hydroxycinnamoyl-CoA shikimate/quinate hydroxycinnamoyl transferase (HCT), 4-coumarate-CoA ligase (4CL), Cinnamoyl-CoA reductase (CCR), cinnamyl-alcohol dehydrogenase (CAD), and peroxidase (POD) are key enzymes in the process of lignin biosynthesis (Tronchet et al., 2010; Xu et al., 2011; Govender et al., 2017). Functional investigation have showed that deactivation of *CAD, CCR*, and *POD* genes could reduce lignin synthesis in various plant species (Tronchet et al., 2010; Xu et al., 2011; Govender et al., 2017). In the present study, these genes were significantly up-regulated in the two genotypes (**Figures 5A, B**). Therefore, it can be inferred that the up-regulation of *HCT, 4CL, CAD, CCR* and *POD* in wheat may potentially contribute to the enhancement of lignin synthesis, thereby playing a noteworthy role in conferring resistance against *Pst*.

Anthocyanin, a crucial secondary metabolite in plants, plays a significant role in various plant developmental processes and responses to biotic stress. It possesses notable antibacterial, antiviral, and fungicidal properties (Dong and Lin, 2021; Lev-Yadun and Gould, 2009). Anthocyanins may contribute to plant defense against pathogenic organisms either directly as chemical repellents or indirectly as visual signals (Lev-Yadun and Gould, 2009). In the current study, key genes encoding chalcone synthase (CHS) and chalcone isomerase (CHI) for anthocyanin synthesis in the flavonoid pathway were induced in both genotypes (**Figures 5A, B**). The expression levels of *CHS* and *CHI* were also increased with *Pst* inoculation time (**Figure 5B**), indicating that these genes may exert a significant influence throughout the entire course of *Pst* infection, particularly in the later stages of resistance, by promoting the accumulation of anthocyanins. According to Himeno et al. (2014), the accumulation of anthocyanin in Arabidopsis has the potential to mitigate leaf cell death resulting from infection by ‘*Candidatus* Phytoplasma asteris’ OY-W strain. Hence, the up-regulation of these crucial genes leading to increased anthocyanin accumulation may serve as an early to late mechanism of *Pst*-resistance in wheat.

### 4.5 Circadian rhythm is associated with the wheat resistance against *Pst* during the later stage of infection

The circadian clock has been increasingly recognized as a crucial component in the defense mechanisms of plants against pathogens and pests (Lu et al., 2017; Zhang et al., 2019; Yamaura et al., 2020). Rhythms are generated by interlocking transcriptional-translational feedback loops in the core oscillator. The primary constituents of the plant circadian system include adagio1 (ADO1), the late elongated hypocotyl/circadian clock associated 1 (LHY/CCA1), gigantea (GI), the evening complex (EC), lux arrhythmo (LUX), E3 ubiquitin-protein ligase (COP), and the pseudo-response regulator (PRR) family (Lu et al., 2017; Zhang et al., 2019; Yamaura et al., 2020). The disruption of certain clock genes, such as CCA1/LHY, leads to reduced resistance against bacterial, oomycete, and fungal pathogens (Yamaura et al., 2020). In Arabidopsis, the circadian clock is responsible for regulating the rhythmic expression of numerous resistance genes associated with innate immunity, thereby enabling the plant to effectively combat the oomycete pathogen *Hyaloperonospora arabidopsidis* and prevent downy mildew (Wang et al., 2011). ADO1, a significant component of the Arabidopsis circadian system, exhibits altered gene expression and cotyledon movement in a loss-of-function ado1 mutant (Jarillo et al., 2001). The transcription factor *HY5*, which functions as a molecular hub in light signal transduction, provides the link between blue-light perception and the circadian clock (Hajdu et al., 2018; Su et al., 2021).

**Figure 6:**
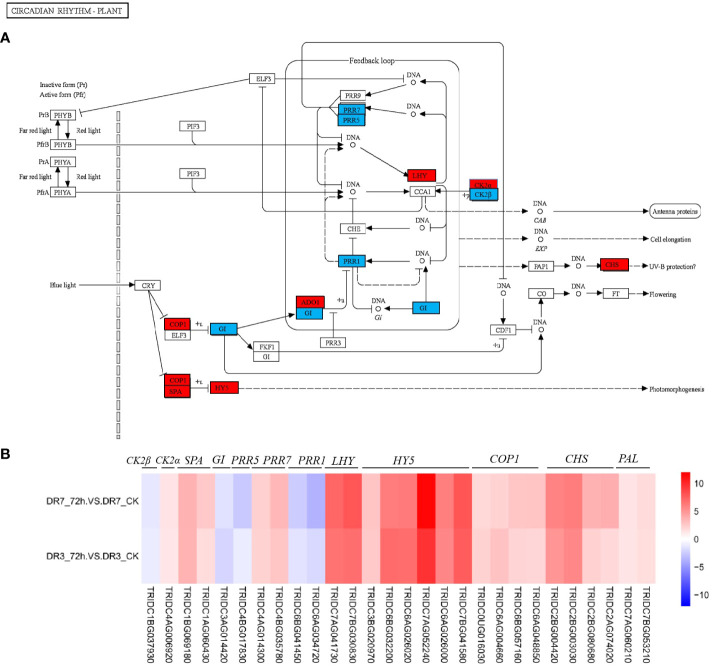
Circadian rhythm pathway and its expression pattern in response to *Pst* infection at 72 hpi in the two wild emmer wheat genotypes, DR7 and DR3. **(A)** The Circadian rhythm pathway map. The regulatory network was constructed based on the expression levels of known genes in this pathway. The blue color signifies downregulation, whereas the red color indicates upregulation at 72 hpi in both the DR7 and DR3 genotypes. **(B)** A heatmap showing fold-changes of *Pst*-responsive genes in circadian rhythm pathway between DR3_72h *vs* DR3_CK, and DR7_72h *vs* DR7_CK. The color gradient represents log_2_fold-change between the *Pst*-infection group and the control group with red color referring to the upregulated genes and blue color referring to the downregulated genes. PAL, phenylalanine ammonia-lyase; CHS, chalcone synthase; COP, E3 ubiquitin-protein ligase; LHY, Late elongated hypocotyl, PRR, Pseudo-response regulator; GI, Gigantea; SPA, suppressor of phytochrome A; CK2α, casein kinase II subunit alpha; CK2β, casein kinase II subunit beta.

Here, *LHY, ADO1, CHS, COP, HY5*, and *SPA* in the pathway of circadian rhythm were significantly upregulated in the two genotypes, while *PRR1* (also known as *TOC1*), *PRR7, PRR5*, and *GI* were significantly downregulated (**Figures 6A, B**). In fact, *CCA1* and *LHY* encode Myb-like transcription factors that have been reported to repress the expression of *PRR7*, *PRR5*, *PRR1* and GI genes (Yamaura et al., 2020; Su et al., 2021). Our data indicate that the circadian clock plays a crucial role in conferring resistance against *Pst* in wild emmer wheat. However, our current understanding of involvement of the circadian clock in defense regulation is still in its early stages, and further research is needed to elucidate the underlying mechanisms. Previous studies have demonstrated that certain clock genes regulate plant defense through a stomata-dependent pathway (Lu et al., 2017). Abnormal expression of clock genes, such as *CCA1*, *LHY* and *TOC1*, has been observed to disrupt the diurnal patterns of stomatal opening and closure (Zhang et al., 2013; Korneli et al., 2014; Lu et al., 2017). In fact, plants have the ability to actively induce stomatal closure as a defense mechanism against pathogen invasion by perceiving the invader as nonself. *CCA1* and *LHY* were shown to impose time-of-day dependence on (gate) the plant response to *P. syringae* induced stomatal closure (Zhang et al., 2013).

### 4.6 Numerous transcription factors response to the *Pst* infection

Numerous prominent plant transcription factor families, such as *AP2-ERF*, *bHLH*, *NAC*, *bZIP*, and *WRKY*, have been recognized for their pivotal involvement in the response to biotic stresses (Viswanath et al., 2023). In this study, it was observed that *WRKY*, *MADs* and *AP2-ERF* families exhibited participation in the initial stage response (24 hpi) to *Pst* invasion, while the *MYB*, *NAC*, *TCP* and *b-ZIP* families contributed to defense during the later stage (72 hpi) of *Pst* infection (**Table 1**, **Figure 4**).

The *AP2/ERF* family of transcription factors holds significant importance in the plant kingdom due to their involvement in growth, development and responses to a range of biotic and abiotic stresses (Moffat et al., 2012; Zheng et al., 2019; Liu et al., 2023a). Numerous *ERFs* have been found to activate the transcription of defense-related genes, including pathogenesis-related (PR) genes, osmotin, chitinase, and β-1,3-glucanase (Moffat et al., 2012; Liu et al., 2023a). Additionally, *ERF11* has been identified as an activator of *BT4* transcription, which plays a crucial role in regulating immunity against *Pseudomonas syringae* (Zheng et al., 2019). In the present study, *ERF1* and *ERF23* were identified as hub genes within the turquoise module. Notably, the expression of these genes exhibited a significant induction at 24 hpi, with a 7.9-fold (log_2_fold-change = 2.95) and 7.5-fold (log_2_fold-change = 2.84) increase in the DR7 genotype. In comparison, the DR3 genotype exhibited a 37.3-fold (log_2_fold-change = 5.36) and 33.6-fold (log_2_fold-change = 5.32) increase in expression (**Figure 4D**). These findings suggest that *ERF1* and *ERF23* play a crucial role in the early stage response of wheat to *Pst* infection, with a more pronounced regulatory impact on the moderately *Pst*-resistant genotype DR3. Additionally, *ERF34* and *ERF43* were identified as hub genes within the blue module, displaying significant upregulation at 72 hpi. Specifically, *ERF34* exhibited a 23.4-fold increase in expression in DR7 and a 10.7-fold increase in DR3, while *ERF43* demonstrated even greater induction with a 41.6-fold increase in DR7 and a 43.1-fold increase in DR3 (**Figure 4D**). These findings strongly imply the potential involvement of *ERF34* and *ERF43* in the immune response of wild emmer wheat to *Pst* infection during the later stages of the response. Consistent with these results, a previous study by Hawku et al. (2021) presented similar findings, demonstrating the positive involvement of TaAP2-15, an *AP2/ERF* transcription factor, in conferring resistance to the stripe rust fungus in wheat.

*WRKY* TFs are a large family of regulators that play crucial roles in various developmental and physiological processes, especially in response to diverse biotic and abiotic stresses. In Arabidopsis, a majority of *WRKY* genes have been identified as pivotal contributors in defense against pathogenic invasions (Mao et al., 2011; Joshi et al., 2022; Ng et al., 2018; Viswanath et al., 2023). Similarly, in rice, the *WRKY* genes showed positive effects in the rice blast fungal interaction (Shimono et al., 2007; Cheng et al., 2015; Liu et al., 2018). In wheat, the transient expression of *HvWRKY6*, *HvWRKY40* and *HvWRKY70* has been observed to enhance the resistance of wheat against *P. triticina* (Gao et al., 2018). In the current study, it was observed that a majority of *WRKY* genes displayed induction at 24 hpi in both the DR7 and DR3 genotypes. Especially, the induction of hub genes, including *WRKY22, WRKY24, WRKY28* and *WRKY50*, exhibited a significant increase of over 120-fold in the comparison of DR3_24 *vs* DR3_CK (**Figure 4D**). These findings suggest that these WRKY TFs play a crucial role in wheat resistance against *Pst* infection during the early stage response. Furthermore, they exert a more pronounced regulatory impact on the DR3 genotype response to *Pst* infection compared to the DR7 genotype. In contrast, the expression of *WRKY19* exhibited an increase over time following *Pst* inoculation, with a greater magnitude of increase in the DR7 genotype compared to the DR3 genotype (**Figure 4D**). This suggests that *WRKY19* may have a substantial involvement in both the early and late responses to *Pst* infection, exerting a particularly pronounced impact in the highly resistant DR7 genotype.

Research on *bZIP* transcription factor has mostly focused on their role in response to abiotic stress and development, but there is increasing evidence that they also play a role in regulating plant immunity against pathogens. For instance, the interaction between *bZIP23* and *NAC28* has been shown to modulate rice resistance against sheath blight disease (Yuan et al., 2023). Likewise, the overexpression of *Vitis vinifera VvbZIP60* has been found to enhance Arabidopsis’s resistance to powdery mildew by activating the salicylic acid signaling pathway (Yu et al., 2019). In wheat, *TabZIP74* functions as a positive regulator in wheat stripe rust resistance. When *TabZIP74* was suppressed through gene silencing induced by barley stripe mosaic virus, the susceptibility of wheat seedlings to stripe rust increased (Wang et al., 2019). In the current study, analysis of the blue module identified *bZIP19* and *bZIP46* as hub genes, which were significantly upregulated at 72 hpi. Specifically, the expression of *bZIP19* was upregulated by 8.7-fold (log_2_fold-change = 3.12), while *bZIP46* showed a 38-fold upregulation (log_2_fold-change = 5.19) in the DR7 genotype (**Figure 4D**). In DR3 genotype, the expression of *bZIP19* was upregulated by 25.9-fold (log_2_fold-change = 4.69), while *bZIP46* exhibited a 19.3-fold upregulation (log_2_fold-change = 4.26) (**Figure 4D**). According to these findings, *bZIP19* and *bZIP46* may be involved in the immune response to *Pst* infection in wild emmer wheat. Additionally, our study successfully identified seven *HY5* genes that displayed significant induction only at 72 hpi, indicating the potential involvement of *HY5* in the late-stage plant immune response to *Pst* infection in wild emmer wheat. *HY5*, a member of the *b-ZIP* family, plays a crucial role in light signal transduction, plant growth and development, and circadian rhythm regulation (Gangappa and Botto, 2016). To date, only one study has provided evidence that *HY5* in Arabidopsis not only serves as a significant element in light signaling regulation, but also actively enhances host plant immunity against *H. arabidopsidis* by transcribing defense-related genes (Chen et al., 2021). Our findings provide additional evidence that supports the essential role of *HY5* in regulating plant immune responses to pathogens.

Although some *Pst*-modulated transcription factors have been identified, few are known in wild emmer wheat. Additionally, the roles of novel identified *Pst*-regulated transcription factors, such as *ERF* and *HY5*, in wheat response to *Pst*-infection are largely unexplored. More work will be carried out to validate transcription factors identified in this study and develop diagnostic molecular markers to accelerate the introgression of the novel resistance gene into new wheat cultivars. Furthermore, with the rapid development of transgenic technology and genome editing technologies, these findings coupled with the technologies will offer novel opportunities for the enhancement of resistance in wheat cultivars.

## 5 Conclusion

In summary, the study aimed to examine the transcriptome profiles of leaves from two wild emmer wheat genotypes with different levels of resistance to *Pst*. Following exposure to *Pst*-infection, numerous genes were significantly induced in wild emmer wheat. The findings revealed a higher number of DEGs in the moderately *Pst-*resistant genotype, while the highly *Pst-*resistant genotype exhibited a greater enrichment of pathways. These results indicate a significant disparity in the response to *Pst* between the two genotypes. Nonetheless, there were consistent patterns in the enrichment of pathways between the two genotypes at the same time of inoculation. The resistance of wild emmer wheat to *Pst* may be linked to the biosynthesis of secondary metabolites, phenylpropanoid biosynthesis, phenylalanine metabolism, alpha-Linolenic acid metabolism, and circadian rhythm-plant. Additionally, numerous transcription factors, such as *ERF1*, *ERF23, WRKY22* and *HY5*, were identified to be involved in the response to *Pst* infection. These findings contribute to a better understanding of the molecular mechanisms underlying the response to strip rust in wild emmer wheat.

## Data availability statement

The datasets presented in this study are publicly available. This data can be found in the SRA database in NCBI (link: https://dataview.ncbi.nlm.nih.gov/object/PRJNA1037698; accession number: BioProject PRJNA1037698).

## Author contributions

JR: Data curation, Funding acquisition, Project administration, Visualization, Writing – original draft, Writing – review & editing. LC: Conceptualization, Methodology, Validation, Writing – review & editing. JL: Data curation, Investigation, Visualization, Writing – original draft. BZ: Software, Visualization, Writing – original draft. YS: Formal analysis, Methodology, Writing – original draft. GH: Data curation, Visualization, Writing – review & editing. JP: Resources, Writing – review & editing.
