# Supplementary figures and images for: Transcriptomic insights into the molecular mechanism for response of wild emmer wheat to stripe rust fungus
Source: Front Plant Sci. 2024 Jan 3;14:1320976. doi: 10.3389/fpls.2023.1320976 (PMC10791934; doi:10.3389/fpls.2023.1320976)

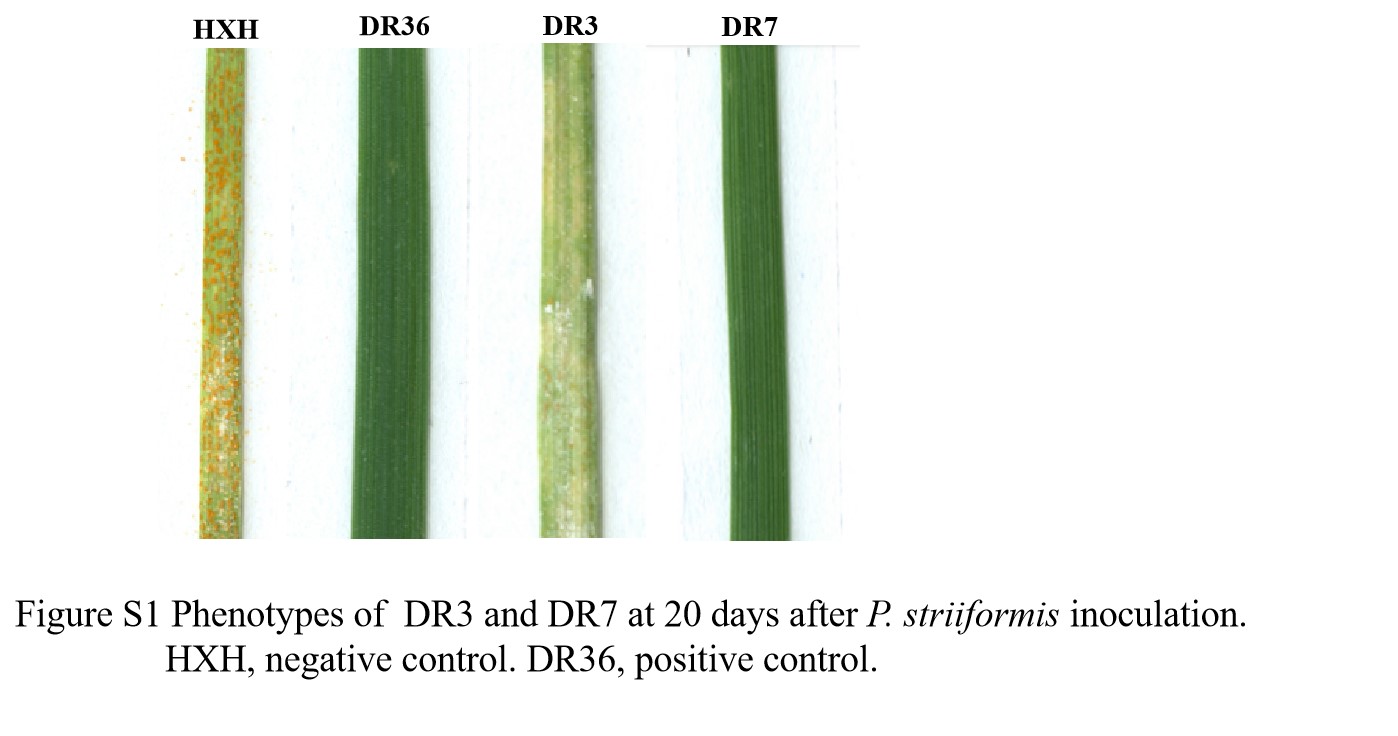

Supplement: Supplementary file 1 [file Image_1.jpeg]

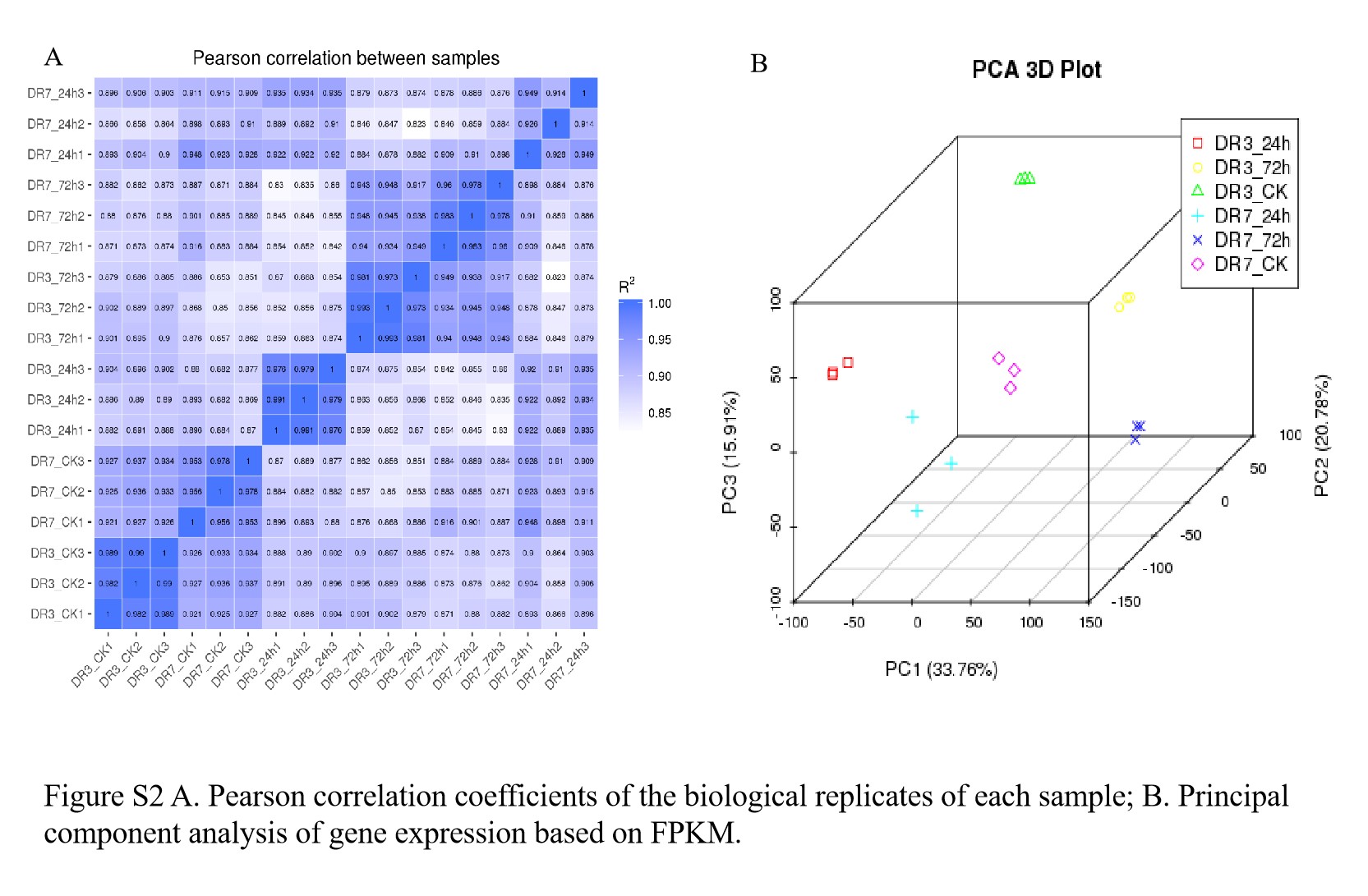

Supplement: Supplementary file 2 [file Image_2.jpeg]

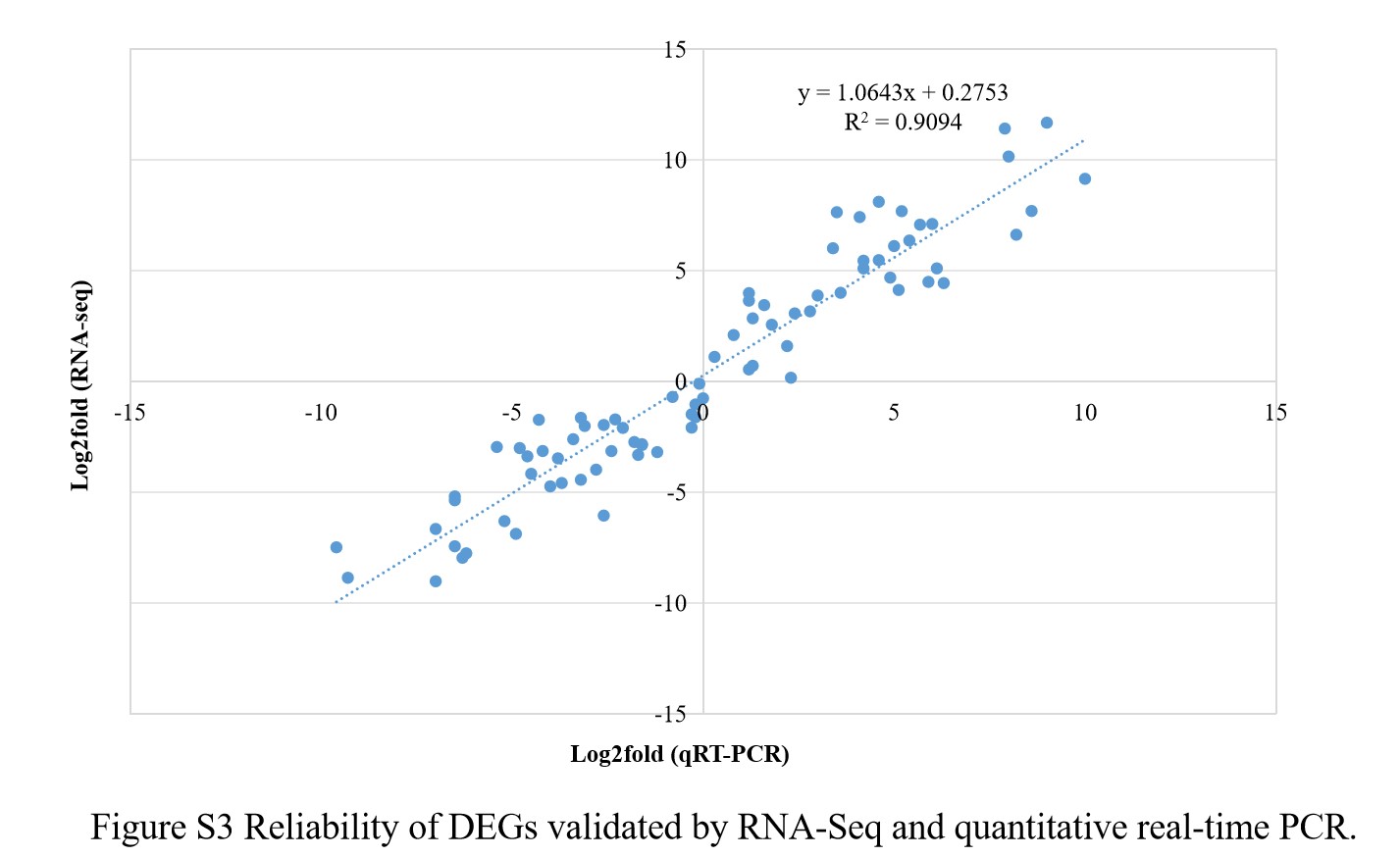

Supplement: Supplementary file 3 [file Image_3.jpeg]
